# Advancing Precision in IBD Care: Innovations in Diagnosis, Monitoring, and Multidisciplinary Management

**DOI:** 10.3390/biomedicines13061421

**Published:** 2025-06-10

**Authors:** Pedro Vilela Teixeira, Fernando Magro, Maria Manuela Estevinho

**Affiliations:** 1 Department of Gastroenterology, Unidade Local de Saúde Gaia Espinho (ULSGE), 4434-502 Vila Nova de Gaia, Portugal; pedrovilelateixeira@gmail.com; 2 Unit of Pharmacology and Therapeutics, Department of Biomedicine, Faculty of Medicine, University of Porto, 4200-319 Porto, Portugal; fm@med.up.pt; 3 Department of Gastroenterology, Unidade Local de Saúde São João (ULSSJ), 4200-319 Porto, Portugal

Inflammatory bowel disease (IBD), encompassing Crohn’s disease (CD) and ulcerative colitis (UC), is a multifactorial disorder involving a dynamic interplay between genetic susceptibility, gut microbiota, nutrition, environmental exposures, immune dysregulation, and psychosocial factors [1,2,3,4]. Despite the growing armamentarium of advanced therapies, the management of IBD continues to face persistent and complex challenges. As global incidence and prevalence of IBD steadily rise [5], clinicians must still contend with diagnostic delays, suboptimal methods of disease monitoring, and limited tools to predict the therapeutic response. Modern IBD management aims to operate beyond symptom control, targeting mucosal and histological healing to reduce hospitalizations, steroid use, and cancer risk [6,7]. The unpredictable nature of disease flares and the diversity of disease phenotypes, combined with the fact that 30–50% of patients fail to respond to first-line biologic therapy [8,9], all highlight the inadequacy of a “one-size-fits-all” approach. There is a clear need for precision medicine strategies that enable individualized treatment plans aimed at preventing long-term complications such as strictures, fistulas, and surgery [10].

This Special Issue brings together a collection of innovative studies and perspectives that explore a range of diagnostic tools, biomarkers, and therapeutic approaches. Together, they represent the ongoing efforts to optimize disease monitoring, improve therapeutic outcomes, and advance toward more personalized standards of care in IBD.

In their contribution to this Special Issue, Pavel and colleagues (Contribution 1) present a prospective study exploring the predictive value of mucosal cytokine expression in response to biologic therapies among patients with CD and UC. The authors highlight a nuanced immunological landscape by analyzing colonic biopsies from patients both before and after 4–6 months of biologic or small-molecule therapy. While TNF-α expression was generally higher in non-responders, it did not significantly distinguish responders from non-responders within the anti-TNF-treated subgroup. Most strikingly, high pre-treatment expression of IL-10, typically an anti-inflammatory cytokine, was associated with a poorer therapeutic response and greater histological activity, whilst high post-treatment IL-10 expression correlated with a longer disease duration. In contrast, responders exhibited lower baseline IL-10 levels and demonstrated a significant increase following treatment, suggesting that IL-10 resistance or dysregulation may underlie treatment failure in a subset of patients. Furthermore, the overexpression of dysplastic markers such as p53 and Ki-67 was associated with features of histological severity and supports the need for enhanced colorectal cancer surveillance in these patients. This study underscores the potential of IL-10 mucosal profiling as a prognostic tool and advances the case for precision medicine approaches in IBD.

The study by Gabriel-Segard et al. (Contribution 2) offers valuable insight into the psychological dimensions of IBD. While fatigue is a widespread complaint among IBD patients—often attributed to active inflammation or extraintestinal manifestations—this study highlights that a significant proportion of patients with quiescent disease also report persistent fatigue. The authors suggest a possible alteration in the dopaminergic pathways involved in reward and motivation, even in the absence of active intestinal inflammation. Moreover, the study raises the compelling hypothesis that the gut–brain axis, potentially modulated by the IBD-associated microbiome, may contribute to these neuropsychiatric changes. This work underscores the need for transdisciplinary approaches to fatigue in IBD and points toward novel therapeutic targets beyond traditional anti-inflammatory or antidepressant strategies.

In an innovative contribution, Dranga and colleagues (Contribution 3) explore the potential of machine learning to enhance the predictive power of fecal calprotectin for assessing endoscopic disease activity (EDA) in patients with UC. The authors conducted a prospective study involving 98 UC patients, integrating fecal calprotectin with clinical and laboratory parameters, including fibrinogen, platelet count, and a clinical disease activity index, into multilayer perceptron (MLP) models. The results were compelling; while calprotectin alone achieved modest predictive performance (AUC 0.68, accuracy 70%) the combined model significantly improved diagnostic accuracy (AUC 0.93, accuracy 85%), outperforming traditional approaches. This study shows machine learning can transform low-cost biomarkers into accurate, non-invasive tools for monitoring UC activity.

In their insightful study, Poenariu et al. (Contribution 4) delve into the role of systemic inflammation in UC by examining serum hypoxia-inducible factor-1 alpha (HIF-1α), along with the following two emerging hematological indices: the mean corpuscular volume to lymphocyte ratio (MCVL) and the cumulative inflammatory index (IIC). HIF-1α, produced by intestinal epithelial cells in response to hypoxia, activates a cascade of protective genes aimed at preserving epithelial barrier integrity. In this prospective study, serum HIF-1α levels correlated significantly with both disease activity and extent, demonstrating its potential as a non-invasive biomarker. The hematologic indices MCVL and IIC also showed promise as they demonstrated diagnostic accuracies of 70.90%, although with a lower specificity compared to HIF-1α.

Lartey et al. (Contribution 5) present a forward-looking review that highlights the transformative potential of fibroblast activation protein inhibitor positron emission tomography/computed tomography (FAPi PET/CT) as an advanced imaging modality for visualizing fibrosis in immune-mediated inflammatory diseases (IMIDs). By targeting activated fibroblasts, FAPi PET/CT enables high-resolution, real-time detection of fibrotic remodeling across organ systems. This technique has demonstrated superior performance to conventional imaging and FDG-PET in detecting early fibrotic changes and distinguishing inflammation from fibrosis. For CD, FAPi PET/CT outperformed CT enterography in identifying mild-to-moderate endoscopic lesions and revealed submucosal fibrotic activity undetectable by standard modalities. This review underscores the emerging clinical value of FAPi PET/CT as it can revolutionize diagnosis, disease monitoring, and therapeutic decision-making in fibrotic complications of IMIDs.

In conclusion, the work presented in this Special Issue reflects a meaningful shift in how we approach IBD, moving beyond the gut to consider the full complexity of the disease. From improved use of familiar biomarkers to cutting-edge imaging and machine learning tools, these studies point toward a future of more precise, less invasive care. What stands out is not a single breakthrough but a collection of thoughtful, incremental advances that together are changing the landscape of IBD research and treatment. As our understanding deepens, so too must our definition of what it means to truly control and monitor this disease.
